# Assessing the knowledge, attitudes and practices of physicians on antibiotic use and antimicrobial resistance in Iran: a cross-sectional survey

**DOI:** 10.1186/s40545-022-00484-2

**Published:** 2022-11-14

**Authors:** Ramin Sami, Raheleh Sadegh, Fataneh Fani, Vajihe Atashi, Hamid Solgi

**Affiliations:** 1 Department of Internal Medicine, School of Medicine, Isfahan University of Medical Sciences, Isfahan, Iran; 2 Resident of Community and Prevention Department, Isfahan University of Medical Sciences, Isfahan, Iran; 3 Infection Control Unit of Amin Hospital, Isfahan University of Medical Sciences, Isfahan, Iran; 4 Adult Health Nursing Department, Nursing and Midwifery Care Research Center, School of Nursing and Midwifery, Isfahan University of Medical Sciences, Isfahan, Iran; 5 Isfahan Endocrine and Metabolism Research Center, Isfahan University of Medical Sciences, Isfahan, Iran; 6 Division of Clinical Microbiology, Department of Laboratory Medicine, Amin Hospital, Isfahan University of Medical Sciences, Isfahan, Iran

**Keywords:** Antibiotic resistance, Antibiotics, Physicians, Knowledge, Attitudes and practices, Iran

## Abstract

**Background:**

Antimicrobial resistance (AMR) is a global public health issue. Physicians should play a key role to fight AMR, and medical education is a fundamental issue to combat it. Understanding the knowledge, attitudes and practices of physicians regarding antibiotic prescription and antibiotic resistance is fundamental for controlling the irrational antibiotic use. This study was conducted to assess the knowledge, attitudes and the practices of physicians in Iran with respect to antibiotic resistance and usage.

**Methods:**

A cross-sectional study was performed from June to October 2021 among physicians at primary care centers and academic hospitals in the region of Isfahan, Iran. A total of 182 physicians were surveyed. Participants were invited to complete a self-reported questionnaire (paper based or online questionnaire). The questions were based on knowledge, attitude, and practice toward antibiotic usage and AMR. Data were analyzed using SPSS version 18 software following the objective of the study.

**Results:**

Out of 182 study participants, 100, 50 and 32 responders were medical doctors (MD), internist and other specialists, respectively. Regarding the knowledge section of the questionnaire, almost less than 10% of participants declared to know the antibiotics of Iran's antimicrobial stewardship program. Also, the percentage of participants who correctly responded to clinical quizzes was 23% for treatment of extended-spectrum beta-lactamase (ESBL) producers, 59.3% about the treatment of severe sepsis, 22% about the intrinsic resistance of *Proteus mirabilis* and 43.4% for experimental treatment with vancomycin in community-acquired pneumonia. Regarding attitude, most participants (97.2%) were aware of the antimicrobial resistance problem in Iran, and 95.6% agreed that prescribing antimicrobials was not the appropriate in our country. Regarding practice, only 65.9% of participants said that before prescribing antibiotics they use of local and international antimicrobial therapy guidelines and less than 50% of physicians were in contact with a microbiology laboratory.

**Conclusion:**

This data revealed that our physicians' level of knowledge about AMR and antimicrobial stewardship is poor, so there is the need to increase training on antibiotic resistance and antimicrobial stewardship.

## Background

Antimicrobial resistance (AMR) has been recognized as a major health priority by the World Health Organization (WHO) [1]. Emergence and spread of AMR is causing not only increased morbidity and mortality, but also a high economic burden [2]. In 2019, an estimated 4.95 million deaths were associated with AMR globally, with 1.27 million deaths being attributed to AMR [3]. With the increasing risk of AMR, estimates that, by 2050, as many as 10.2 million people will die every year, 90 percent of which are expected to burden Africa and Asia [4]. There is a consensus that the irrational use of antibiotics has contributed to the problem of AMR. Unnecessary or inappropriate empiric antibiotic therapy and over-prescription of antibiotics are prevalent worldwide [5, 6]. A study published in 2021 reported that there was a 46% increase in the global volume of antibiotic consumption between 2000 and 2018. This study [7] also revealed that global antibiotic consumption rates increased from 9.8 daily defined doses (DDD) per 1000 per day in 2000 to 14.3 in 2018 [7].

According to the WHO, the total consumption of antibiotics in in Iran was 38.8 DDD per 1000 inhabitants per day in 2015. This was the highest consumption reported in the WHO Eastern Mediterranean Region [8].

Several factors may contribute to irrational antimicrobial usage, including physicians’ knowledge, attitudes and practices, uncertain diagnosis, and patients’ expectations [9]. Healthcare workers also play important roles in minimizing antimicrobial misuse through interventions such as implementation of infection control programs [10]. The knowledge, attitudes and prescribing (KAP) behavior of physicians plays a critical role in the consumption of antibiotics and is a potential tool for control AMR. In addition, medical students and interns are an important target group for sustainable antibiotics prescribing intervention measures [2]. There is only little KAP-information among health care workers such as physicians, medical students, or pharmacists in healthcare settings compared to a wider variety of community-based studies [11, 12]. In addition, the available studies have been performed among physicians in healthcare settings, and these are mostly from the Americas and Europe [13–15].

In Iran, studies have shown high levels of antibiotic resistance in healthcare settings [16–18], but reports assessing the KAP of physicians towards AMR are not available. Despite the lack of data, available evidence implies that awareness physicians should be addressed. We therefore conducted the first Iranian KAP-survey among physicians in hospitals in Isfahan with respect to antibiotic resistance and usage. The objective of this study was to assess knowledge, perceptions, and attitudes in relation to antibiotic prescribing among physicians practicing in hospitals in Isfahan, Iran.

## Materials and methods

### Study design and setting

In the period from 27 June to 17 October 2021, a cross-sectional, multicenter, paper and online-based survey was conducted in academic hospitals in the region of Isfahan, Iran. The sample size was calculated using the formula:

$$N = \frac{{Z_{{1 - \alpha /_{2} }}^{2} \cdot P\left( {1 - P} \right)}}{{d^{2} }}.$$

*N* is the number of participants. Where anticipated *P* = 0.25, *α* = 5%, *D* = 0.7%,

$$N = \frac{{\left( {1.96} \right)^{2} \times 0.25 \times 0.75}}{0.0049}.$$

The calculated sample size was 147. In this study, we invited 235 physicians and 182 of them answered the questions completely.

### Participants

Iranian physicians aged from 25 to 65 years, including medical doctors (MD), internist and various clinical specialties (neurology, general surgery, urology, gynecologist, anesthesiologist, orthopedist and emergency medicine) were included from the study. Initially, participants were invited to complete a self-reported questionnaire with paper based, due to a low response rate, an online questionnaire was used in the second stage. We collected the participants’ demographics and general information data, including age, gender, professional status and specialty.

### Questionnaire design

The questionnaire used in this study was developed in consultation with experts on medical bacteriology, and after searching the literature for similar studies [2, 15, 19]. Before the main survey, a small-scale pilot study was conducted on 10 physicians from two primary care facilities. Based on the pilot study, the questionnaire was revised and removal of some items. The final version of the questionnaire had 23 questions subdivided into three sections: (i) knowledge related to awareness about antimicrobial stewardship in Iran, concepts of drug sensitivity and susceptibility, side effects of antibiotics, relationship of disease, antimicrobial use and AMR, including clinical quizzes on appropriate management of specific infections [methicillin-resistant *Staphylococcus aureus* (MRSA)], vancomycin-resistant enterococci (VRE), extended-spectrum β-lactamase-producing enterobacteria (ESBL), and carbapenem-resistant Enterobacteriaceae (CRE); (ii) attitudes associated with harmful effects of antibiotics, seriousness of antibiotic abuse and perceptions about the relevance of the AMR issue; (iii) practices related to participants’ antibiotics knowledge, awareness of the pattern of AMR at the hospitals where the work is done, factors influencing antibiotic prescription, use of international and local guidelines, consultation with infectious disease specialists and communication with microbiology laboratory.

### Data analysis

Data were analyzed by using SPSS version 18 software following the objective of the study. Qualitative variables were presented by frequency and percentage and we applied Chi-square and Fisher’s exact test for analysis of potential associations of knowledge, attitudes and perception with participants’ age, gender, professional status and specialty.

## Results

### Participants

Among the 235 physicians who were invited, 182 (77.4%) fully completed the questionnaire and were included in the analysis. The mean age of respondents was of 33.2 years and 105 (57.7) were female. Most of the respondents, 93 (51%) were within the age group 31–40 years. The largest group of respondents was MD 100 (54.9%), followed by internist 50 (27.5%), and other specialists 32 (17.6%). A total of 128 (70.3%) respondents had < 5 years practice experience, whiles 39 (21.4%) had practiced for 5–15 years and 15 (8.3%) for ˃ 15 years. The demographic characteristics of the study participants are summarized in Table 1.

**Table 1:** Demographic characteristics of participants (*n* = 182)

Variables	Number (%)
Age (years)	
24–30	54 (29.8)
31–40	93 (51)
˃ 40	35 (19.2)
Gender	
Male	77 (42.3)
Female	105 (57.7)
Professional status	
MD	100 (54.9)
Internist	50 (27.5)
Other specialists	32 (17.6)
Years of experience	
< 5	128 (70.3)
5–15	39 (21.4)
> 15	15 (8.3)

### Knowledge

In this study, there were ten knowledge questions assigned (Table 2). Regarding the knowledge section of the questionnaire, almost all the participants declared to unknown the kind of antibiotics that categorized into the Iranian antimicrobial stewardship program. In particular, 70.9% of the participants chose I do not know option and only 9.9% answered correctly. Almost one-fifth (23%) of respondents correctly answered to treatment of infections caused by ESBL-producing *Enterobacterial species, less than 10% of respondents correctly replied to question about* prescribing antibiotics for surgical prophylaxis. Less than half (49.5%) and 35.2% of respondents correctly replied to questions about antibiotic permeability in the cerebrospinal fluid barrier and how to administer aminoglycoside antibiotics, respectively.

**Table 2:** Knowledge questions regarding antimicrobial use and resistance and results

No. question and possible answers (correct answer in bold)	Overall (*N* = 182)			MD (*N* = 100)			Internist (*N* = 50)			Other specialists (*N* = 32)		
	Correct *N* (%)	Incorrect *N* (%)	I don’t know [*N* (%)]	Correct *N* (%)	Incorrect *N* (%)	I don’t know [*N* (%)]	Correct *N* (%)	Incorrect *N* (%)	I don’t know [*N* (%)]	Correct *N* (%)	Incorrect *N* (%)	I don’t know [*N* (%)]
1. Which one of the following antibiotics is part of the antimicrobial stewardship program in Iran? **(a) Voriconazole** (b) Ganciclovir (c) Piperacillin/tazobactam (d) I don't know	18 (9.9)	35 (19.2)	129 (70.9)	1 (1%)	10 (10)	89 (89)	12 (24)	16 (32)	22 (44)	5 (15.6)	9 (28.1)	18 (56.2)
2. Which one of the following is not true in case of extended-spectrum beta-lactamase-producing Enterobacteriaceae (ESBL)? (a) The antibiotic of choice for severe infections with these organisms is a carbapenem (b) In mild infections, combined beta-lactam/beta-lactamase inhibitors may be used **(c) In cases of cystitis, third-generation cephalosporins can be used** (d) I don’t know	42 (23)	96 (52.7)	44 (24.2)	16 (16)	50 (50)	34 (34)	17 (34)	26 (52)	7 (14)	9 (28.1)	20 (62.5)	3 (9.4)
3. Which one of the following is true when prescribing antibiotics for surgical prophylaxis? (a) Antibiotics should usually be given 2 h before surgery **(b) Vancomycin and ciprofloxacin should be given 1 to 2 h before surgery** (c) Ceftazidime is preferable to cefazolin in most surgeries (d) I don’t know	18 (9.9)	141 (77.5)	23 (12.6)	7 (7)	80 (80)	13 (13)	3 (6)	40 (80)	7 (14%)	8 (25)	21 (65.6)	3 (9.4)
4. Which one of the following antibiotic most effectively crosses the blood–brain barrier? (a) Vancomycin **(b) Ceftriaxone** (c) Clindamycin (d) I don’t know	90 (49.5)	72 (39.5)	20 (11)	45 (45)	43 (43)	12 (12)	30 (60)	17 (34%)	3 (6)	15 (46.9)	12 (37.5)	5 (15.6)
5. Aminoglycosides such as gentamicin are very active if they are administered as follows: **(a) Parenteral once daily** (b) Orally three times daily (c) Parenteral three times daily (d) I don’t know	64 (35.2)	83 (45.6)	35 (19.2)	36 (36)	37 (37)	27 (27)	18 (36)	28 (56)	4 (8)	10 (31.3)	18 (56.2)	4 (12.5)
6. To start treatment of a patient with sever sepsis, vancomycin and meropenem are prescribed to cover which organisms, respectively? **(a) MRSA-ESBL** (b) MRSA-VRE (c) VRE-CRO (d) I don't know	108 (59.3)	39 (21.4)	35 (19.2)	56 (56)	21 (21)	23 (23)	39 (78)	7 (14)	4 (8)	13 (40.6)	11 (34.4)	8 (25)
7. *Proteus mirabilis* is intrinsic resistant to which of the following antibiotic? **(a) Colistin** (b) Ampicillin/sulbactam (c) Amikacin (d) I don't know	40 (22)	55 (30.2)	87 (47.8)	12 (12)	27 (27)	61 (61)	20 (40)	17 (34)	13 (26)	8 (25)	11 (34.4)	13 (40.6)
8. Which one of the following is true in case of *A. baumannii* which is resistant to all of antibiotics with the exception of colistin? **(a) Extensively drug-resistant** (b) Pandrug resistant (c) Not-multidrug resistance (d) I don’t know	79 (43.4)	16 (8.8)	87 (47.8)	39 (39)	4 (4)	57 (57)	32 (64)	5 (10)	13 (26)	8 (25)	7 (21.9)	17 (53.1)
9. Which one of the following antibiotics has the best activity against anaerobes? (a) Ciprofloxacin (b) Cotrimoxazole **(c) Metronidazole** (d) I don’t know	151 (83)	23 (12.6)	8 (4.4)	81 (81)	15 (15)	4 (4)	43 (86)	5 (10)	2 (4)	27 (84.4)	3 (9.4)	2 (6.2)
10. Which one of the following is not an important risk factor for initiating experimental treatment with vancomycin in community-acquired pneumonia? (a) Known colonization or prior infection with MRSA (b) Gram-positive cocci in clusters on good-quality sputum Gram stain **(c) Gram-negative bacilli seen on good-quality sputum Gram stain** (d) I don't know	79 (43.4)	56 (30.8)	47 (25.8)	30 (30)	35 (35)	35 (35)	34 (68)	11 (22)	5 (10)	15 (46.9)	10 (31.2)	7 (21.9)

The correct answers are in bold

Further, approximately half (59.3%) of the participants answered correctly to the appropriate choice of antibiotic to cover the bacterial agents such as ESBL *producers* and MRSA in case of severe sepsis and only 22% of the respondents answered correctly about the intrinsic resistance of *Proteus mirabilis* to colistin. Less than half (43.4%) of respondents correctly replied so to the question about extensively drug-resistant (XDR) *A. baumannii*. The majority (83%) of respondents correctly answered to select the appropriate treatment in anaerobic infections acquired by patients and only 43.4% of respondents were aware of the important risk factors for administering vancomycin in community-acquired pneumonia (CAP).

### Attitudes

Most 97.2% (*n* = 177) of the respondents agreed or strongly agreed that AMR was an issue of concern in Iran while 95.6% (*n* = 174) agreed that prescribing antimicrobials was not the appropriate in our country (Table 3). Likewise, most (87.5%) respondents agreed or strongly agreed that antibiotics are overused in their workplace hospital. The majority (89%) of the respondents agreed or strongly agreed can play an effective role in a rational antimicrobial stewardship program. Overall, 98.3% of the respondents believed that inappropriate antibiotics prescribing did put patients at risk of developing antimicrobial resistance, 180 (98.9%) agreed or strongly agreed that abuse and overuse of antibiotics has become the main cause leading to bacterial resistance and 177 (97.2%) agreed or strongly agreed that antibiotic resistance affects the health of them and their families.

**Table 3:** Attitudes of respondents about antibiotic prescriptions and the importance of antibiotic resistance

	Strongly agree [*N* (%)]	Agree [*N* (%)]	Neither agree nor disagree [*N* (%)]	Disagree [*N* (%)]	Strongly disagree [*N* (%)]
1. Antibiotics resistance has become a problem in Iran	133 (73)	44 (24.2)	5 (2.7)	0 (0)	0 (0)
2. Antibiotics are used inappropriately in Iran at present and alarming resistance rates	117 (64.3)	57 (31.3)	2 (1.1)	4 (2.2)	2 (1.1)
3. Excessive or inappropriate antimicrobial use in the hospital that I am working now	42 (23.1)	80 (44)	24 (13.2)	31 (17)	5 (2.7)
4. I can play an effective role in a rational antimicrobial stewardship program	68 (37.4)	94 (51.6)	9 (4.9)	10 (5.5)	1 (0.5)
5. Improper prescribing of antibiotics puts patients at risk	124 (68.1)	55 (30.2)	3 (1.6)	0 (0)	0 (0)
6. Improper use of antibiotics has become the main cause leading to bacterial resistance	152 (83.5)	28 (15.4)	2 (1.1)	0 (0)	0 (0)
7. Antibiotic resistance affects you and your family’s health	144 (79.1)	33 (18.1)	3 (1.6)	2 (1.1)	0 (0)

### Practice

Regarding practice, nearly three-quarters (65.9) of respondents said that before prescribing antibiotics they use of local and international antimicrobial therapy guidelines. Nearly three-quarters (76.4%) of respondents said they seek advice from an infectious disease specialist before prescribing broad-spectrum antibiotics. More than half (59.9%) of respondents agreed that under the pressure of colleagues or acquaintances, they prescribe broad-spectrum antibiotics for patients. Additionally, 63.7% of respondents said that they sent a microbial culture test if patients have symptoms of a bacterial infection before prescribing antibiotics. Also, most respondents (151, 83%) agreed that they were aware of the antimicrobial resistance rates and patterns and common organisms in their hospitals. Less than 50% of physicians were in contact with a microbiology laboratory to prescribe the appropriate antibiotic for patients (Table 4).

**Table 4:** Practices of participants regarding use of antibiotics

	Overall (*N* = 182)		MD (*N* = 100)		Internist (*N* = 50)		Other specialists (*N* = 32)	
	Yes *N* (%)	No *N* (%)	Yes *N* (%)	No *N* (%)	Yes *N* (%)	No *N* (%)	Yes *N* (%)	No *N* (%)
1. Before prescribing an antibiotic, I consult local and international antimicrobial therapy guidelines	120 (65.9)	62 (34.1)	56 (56)	44 (44)	42 (84)	8 (16)	22 (68.8)	10 (31.2)
2. I consult with infectious diseases experts to prescribe of broad-spectrum antibiotics	139 (76.4)	43 (23.6)	64 (64)	36 (36)	43 (86)	7 (14)	32 (100)	0
3. Sometimes, under the pressure of colleagues or acquaintances, I prescribe broad-spectrum antibiotics to the patient	109 (59.9)	73 (40.1)	66 (66)	34 (34)	35 (70)	15 (30)	8 (25)	24 (75)
4. I *order* a *bacteria culture* test if patient have symptoms of a *bacterial infection before prescribing antibiotics*	116 (63.7)	66 (36.3)	40 (40)	60 (40)	46 (92)	4 (8)	30 (93.8)	2 (6.2)
5. I start empirical therapy based on patterns of antibiotic resistance and common organisms at my hospital	151 (83)	31 (17)	78 (78)	22 (22)	44 (88)	6 (12)	29 (90.6)	3 (9.4)
6. I am in contact with my local microbiology laboratory to determine and select the appropriate antibiotic panel	88 (48.3)	94 (51.7)	34 (34)	66 (66)	26 (52)	24 (48)	28 (87.5)	4 (12.5)

## Discussion

To the best of our knowledge, this is the first study to assess knowledge, attitudes and practices among physicians with respect to antimicrobial use and resistance in Iran. Antimicrobial stewardship programs have shown to reduce the emergence of antimicrobial resistance and health-care-associated infections [19–21]. Many countries worldwide have developed and are implementing their national action plans on antimicrobial resistance [2, 6, 22], in which antimicrobial stewardship is a key priority. There is also an antimicrobial stewardship program in Iran that includes eight antibiotics, including carbapenems (meropenem/imipenem), colistin, *teicoplanin*, *vancomycin*, linezolid, *caspofungin*, *amphotericin B*, voriconazole. Notably, only 9.9% of our participants correctly answered to antimicrobial stewardship question. This study revealed low levels of antimicrobial stewardship knowledge in the study participants. With regard to low level of participants' awareness of the stewardship program, we suggest that antimicrobial stewardship courses should be introduced into the curriculum of final year medicine programs. It is also recommended that we raise the awareness of physicians by introducing more lectures on antimicrobial stewardship.

The low levels (23%) of physicians who knew about ESBL is worrying since approximately %50 of all *E. coli* and *K. pneumonia* isolated in our hospitals in Iran are ESBL producers [23]. This low percentage of awareness about ESBL could be due to lack of knowledge regarding mechanisms of antibiotic resistance. Labi et al. in a KAP survey on antibiotics resistance among 159 physicians in Ghana 2015, reported that 52% of senior physicians who expressed knowledge about ESBLs [9]. A study conducted in Italy reported that only 32% of participants correctly answered the questions on ESBLs [15].

To assess knowledge on the awareness of intrinsic resistance among bacteria, physicians were asked about the intrinsic resistance of *Proteus mirabilis*. The fact that almost one-fifth of respondents recognized that *P. mirabilis* is inherently resistant to colistin indicates the low level of physicians' knowledge of the mechanisms of antibiotic resistance. Lack of adequate training during medical degree course may be one of the reasons for that [9, 15]. So, this study suggests greater emphasis on education on AMR required for physicians during their university studies.

Recent CDC data revealed that in the United States, 79% of all patients with CAP were treated inappropriately in the hospital setting [24]. A study conducted by Shan et al., reported that among 52 patients with CAP who were treated with vancomycin for a median of 2 days of therapy only 21% (11/52) of patients had risk factors warranting vancomycin empiric therapy [25]. In this study, 43% of respondents correctly answered the question of awareness about the risk factors for initiating experimental treatment with vancomycin in CAP. Interestingly, 30.8% of our participants were not aware that vancomycin is an antibiotic for the treatment of Gram-positive bacteria, not Gram-negative. These findings highlight the urgent need for carefully planned education and training programs to address the knowledge of our physicians about the AMR in Iran.

Similar to physicians from other parts of the world [15, 19, 22], our participants (97.2%) were aware of the growing problem of antimicrobial resistance at local levels. In this study, majority of participants (95.6%) agreed or strongly agree with inappropriate antimicrobial use and alarming resistance rates in Iran. Similar findings have been reported by many studies [9, 19].

Improper use of antimicrobials is a known driver of resistance and most respondents (98.9%) considered excessive or inappropriate antimicrobial prescribing and non-prudent use of antimicrobials as the most important causes of antimicrobial resistance in Iran. This also confirms that physicians are aware of the specific rates of excessive and inappropriate antimicrobial use and antimicrobial resistance in Iran. The findings of this study are in accordance with previous studies in Italy and Greece [15, 19].

Most of our participants (83%) were aware of the patterns of antibiotic resistance and common organisms in their own institutions, and almost half of the participants (51.7%) declared to contact their workplace microbiology laboratory to select the appropriate antibiotic to prescribe. We believe that the lack of communication between the physicians and the microbiology laboratory can lead to incorrect prescription of antibiotics, which ultimately leads to increased antibiotic resistance.

The presence of both local and international antibiotic guidelines can play a very important role in the selection of appropriate antibiotics by physicians. Such guidelines would help rationalize physicians' practice in relation to antibiotics use [2, 22]. Approximately two-thirds (65.9) of respondents declared that they used local and international guidelines before prescribing antibiotics.

As much as 23.6% respondents declared that they prescribe of broad-spectrum antibiotics without consulting the infectious diseases expert. Interestingly, 36 out of 100 MD physicians say that they do not consult infectious disease specialists for prescribing broad-spectrum antibiotics. Improper administration of broad-spectrum antibiotics by MD physicians increases antibiotic resistance.

### Limitations

The main limitation of our study was the relatively small number of participants selected from one province of central Iran which might not reflect the real situation of KAP of physicians in Iran as a whole. A larger sample size would have been better to provide a more generalizable result. Another limitation was the Internet access required to complete the questionnaire. Most of study participants (81%) received the questionnaire link via the Internet that could have led to selection bias. However, this is less likely to affect our results, particularly as the survey was sent to physicians (literate population) who are increasingly using the internet in their practice.

## Conclusion

This is the first study exploring KAP regarding antimicrobial use and AMR in Iran, which is a country with excessive use of antimicrobials and high rates of antimicrobial resistance. Findings from this survey revealed that our physicians' level of knowledge about AMR and antimicrobial stewardship is poor. To improve antibiotic use and control of antibiotic resistance in our hospitals in Iran, there is the need to increase training on antibiotic resistance and antimicrobial stewardship among physicians in the last year of medicine.

## Data Availability

All data generated or analyzed during this study are included in this article.

## Abbreviations

AMR: Antimicrobial resistance
MD: Medical doctors
ESBL: Extended-spectrum beta-lactamase
WHO: World Health Organization
KAP: Knowledge, attitudes and prescribing
MRSA: Methicillin-resistant *Staphylococcus aureus*
VRE: Vancomycin-resistant enterococci
CRE: Carbapenem-resistant Enterobacteriaceae
XDR: Extensively drug-resistant
